# The effects of leg preference and leg dominance on static and dynamic balance performance in highly-trained tennis players

**DOI:** 10.1371/journal.pone.0259854

**Published:** 2021-11-11

**Authors:** Žiga Kozinc, Nejc Šarabon

**Affiliations:** 1 University of Primorska, Faculty of Health Sciences, Izola, Slovenia; 2 University of Primorska, Andrej Marušič Institute, Koper, Slovenia; 3 InnoRenew CoE, Human Health Department, Izola, Slovenia; 4 S2P, Science to Practice, Ltd., Laboratory for Motor Control and Motor Behavior, Ljubljana, Slovenia; University of Innsbruck, AUSTRIA

## Abstract

In this study, 90 (51 males, 39 females) tennis players performed single-leg quiet stance and single-leg landing tasks. For the static standing task, center-of pressure (CoP) velocities, amplitudes, frequency and area were calculated. For the landing tasks, time to stabilization as well as dynamic postural stability index were considered. The analysis of differences between the legs was done based on two methods for a priori determination of leg preference, one based on the preference of kicking a ball and one based on the preference for single-leg jumping. An additional analysis was done based on the leg dominance (determined post hoc), based on the observed performance of the tasks. In case of the classification based on kicking a ball, there was a statistically significantly lower CoP anterior-posterior velocity and anterior-posterior amplitude in static balance task (p ≤ 0.017; 0.17 ≤ d ≤ 0.28) for the preferred leg. The CoP frequency was higher in the preferred leg for both directions (p ≤ 0.002; 0.10 ≤ d ≤ 0.22). For the landing task, CoP medial-lateral time to stabilization was statistically significantly shorter for the preferred leg (0.28 ± 0.38 s) compared to the non-preferred leg (0.47 ± 0.60 s) (p = 0.012; d = 0.38). There were no differences between the legs for the landing task. Moreover, there were no differences between the legs when we used the preference based on jumping for either of the tasks (d ≤ 0.14). The differences between legs in terms of observed dominance were larger than the differences based on the preference, which stresses the need for clear distinction of limb preference and limb dominance in research and practice. Regarding the effect of leg preference, small differences in static balance may exist between the legs (when the preference is based on kicking a ball).

## Introduction

Assessment of postural balance is routinely performed in athletes in order to assess the risk of injuries [1] and also in relation to athletic performance [2]. Stability underlying quiet standing or sitting is called steady-state or static balance, whereas active/dynamic balance refers to the organization of movement strategies for recovery of stability after perturbations (e.g., surface translations) [3]. The latter is further subdivided to reactive and proactive (anticipatory) stability [3]. One of the most common methods to assess static balance is through quantification of center-of-pressure (CoP) movement during quiet stance. Variables related to CoP movement are highly sensitive athletic injuries [4] and may discriminate individuals participating in different sports [5]. In athletes, testing the static balance during single-leg quiet stance testing is the most useful, as parallel stance testing is not challenging enough for this population [5]. Moreover, single-leg testing is necessary to assess the inter-leg differences (e.g. between injured and uninjured leg in athletes [1, 4]). Furthermore, since athletic injuries occur during dynamic conditions, several authors have proposed alternative reactive balance test for a more ecologically valid and practically relevant assessment. For instance, many injuries occur during landing tasks [6, 7]. Indeed, the assessment of stabilization after landing via CoP metrics is related to injury risk [8, 9] and musculoskeletal deformities [10, 11] in athletes. Thus, a combination of quiet stance body sway and stabilization after landing could be viable for comprehensively (i.e., involving static and reactive balance) assessing balance in athletes. A recent systematic review has demonstrated very little common variance between different aspects of balance performance [12], highlighting high task-specificity within the balance/stability domain.

When the injured leg is compared to a non-injured leg, factors other than injury could affect inter-leg differences, and should thereby be considered for appropriate interpretation of the results. One of these factors, leg dominance/preference, has been recently systematically reviewed [13]. The authors identified 46 studies that assessed balance by single-leg quiet stance body sway, landing test and various field balance tests. Their analyses revealed that for most of the outcome parameters, there were no differences between the legs. However, it has to be noted that the majority of the parameters were pooled from a limited number of studies (i.e., 1-3). Moreover, only three studies investigated the effects of leg dominance/preference in landing task. Finally, as also stressed by the authors of the systematic review [13], no consensus exists regarding the appropriate method to determine leg dominance/preference. In athletes, the preferred leg is commonly determined as the leg that the participant would use to kick a ball [5, 14, 15], which corresponds almost perfectly to handedness (the side of the preferred leg for writing and eating) [16]. However, other approaches, such as self-reported preferred leg for single-leg vertical jumping [14, 17], have also been suggested. Due to the similarity of single-leg jump and single-leg landing, this approach could reveal clearer effects of leg preference in landing. Finally, it has to be stressed that preference and dominance are not the same notion [18, 19]. In this article, we will refer to the preferred leg as the self-reported leg that an individual identifies as preferred for a given task, and we will refer to the dominant leg as the leg that is observed to exhibit superior performance. In some tasks, such as kicking a ball, there is an almost perfect agreement between leg preference and dominance, while this might not be the case for other tasks [18].

The purpose of this study was to assess the effects of leg preference and dominance on postural balance during single-leg quiet stance and single-leg landing test, on a convenience sample of highly-trained tennis players. We determined the leg preference using two different approaches (i.e., kicking a ball and single-leg jumping) to investigate whether the method of preference determination affects the inter-leg differences. According to the available literature [13, 16, 17], we hypothesized that trivial to small differences between the legs will be detected in body sway tasks, regardless of the method of preference determination. Our second hypothesis was that larger differences will be confirmed for landing tasks when the leg preference will be determined based on single-leg jumping (in contrast to classification based on the preference to kick a ball). Then, we determined the leg dominance based on observed performance in body sway and landing tasks (separately). We hypothesized that the inter-leg differences will be higher when the legs are classified based on dominance instead of preference. A secondary purpose was to assess the gender effects on postural balance in body sway and landing tasks. No hypothesis was made regarding the effects of gender, as the previous literature is highly contradicting in this respect [20–24]. The rationale for the study was that the effect of leg preference and/or dominance could affect inter-limb comparisons, be it in cross-sectional or longitudinal research or in practice (e.g., comparing the injured/affected limb with the uninjured/affected limb, or screening risk of injuries or falling).

## Methods

### Participants

For this study, 90 tennis players playing on a regional (n = 79) and national (n = 11) level were recruited (51 males, age: 18.4 ± 14.8 years, body height: 177.6 ± 8.7 cm, body mass 68.2 ± 10.6 kg and 39 females, age: 16.2 ± 2.68 years, body height: 169.1 ± 6.7 cm, body mass 61.2 ± 7.7 kg). The participants were well trained, with mean years of training experience of 10.5 ± 7.3 (males) and 7.7 ± 3.8 (females). The self-reported typical frequency of training was 6.3 ± 2.9 sessions/week in males and 6.4 ± 3.3 session/week in females. The participants had to be injury-free in the past 6 months, and exhibit no neurological or chronic non-communicable diseases. The participants were informed about the testing procedures and had signed written informed consent prior to the onset of the experiment. For underage participants, their parents or legal guardians were also informed and signed the consent on their behalf. The experimental protocol was approved by Republic of Slovenia National Medical Ethics Committee (approval no. 0120-99/2018/5) and was conducted in accordance with the Declaration of Helsinki.

### Procedures

**Fig 1:**
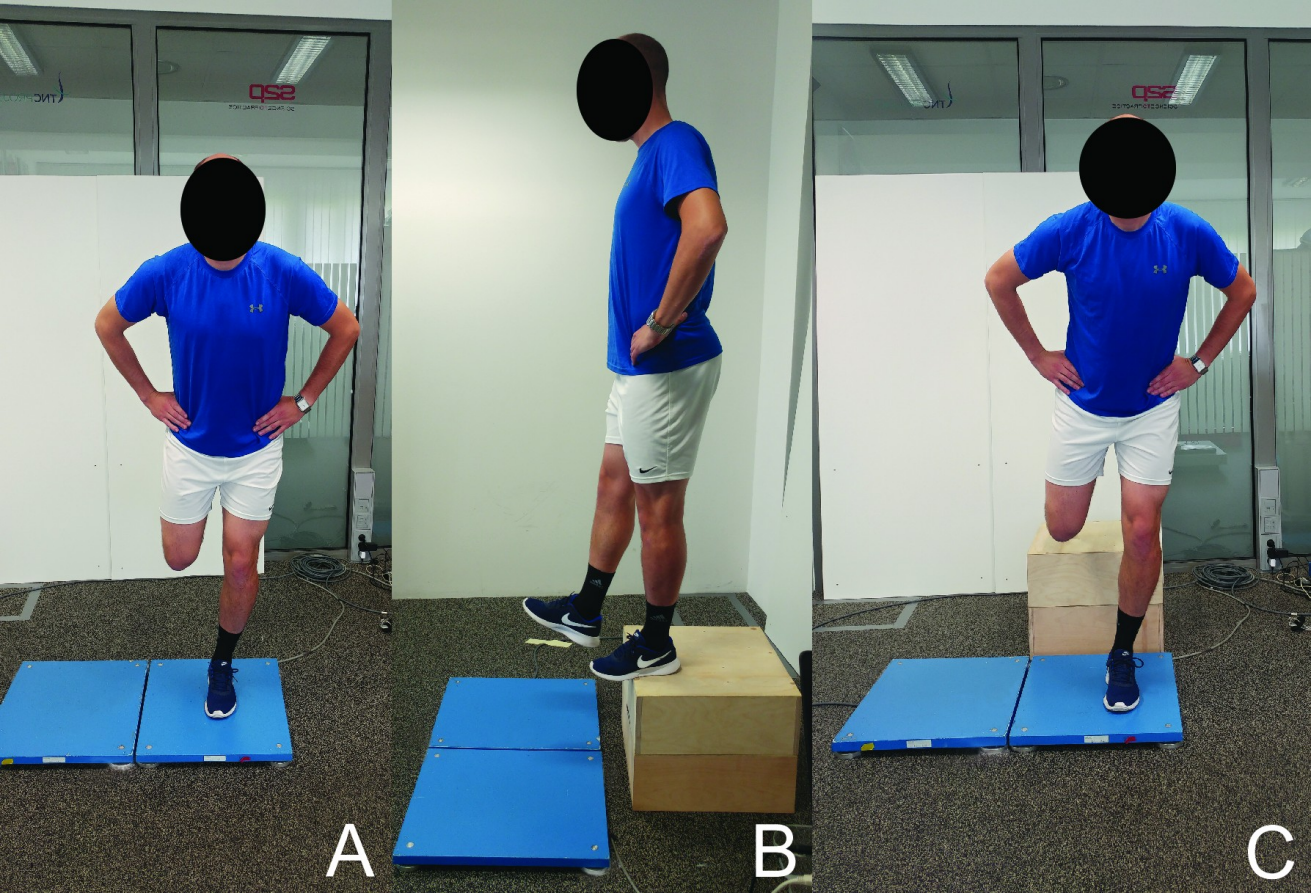
Body sway (A) and landing task (B, C). Note that the second force plate was not used in this study. Participants always performed the tasks on the same force plate to avoid error due to inter-device differences.

The static balance was assessed by quantifying body sway via CoP movements during quiet single-leg stance (Fig 1A). The hip of the opposite (i.e. non-standing) leg was kept in anatomical position (0°) and the thigh had to be parallel to the standing leg, and the knee was bent to 90°. The knee of the standing leg was in the extended position, however, a careful instruction to participants was given not to hyperextend (lock) the joint. The participants were instructed to direct their gaze at a fixed point (black dot on a white background), which was set at an approximately eye level and ~4 m away from the participant. The hands were placed on the hips. Participants performed three 30-s trials with each leg and 60-s breaks were provided between trials. For each trial, the participants acquired the single-leg position, and the examiner started the data acquisition after ~1 s. Both legs were examined in an alternating order across trials, while the starting leg was determined randomly for each participant.

The dynamic balance was assessed with a landing task, wherein the participants were standing on a 40 cm high solid wooden box. They were instructed to stand upright, place their hands on hips, and look straight ahead. Then, they initiated the task with the tested leg by lifting it off the surface and placing it over the edge of the box (Fig 1B) and then dropping it down to land in a single-leg stance on a force plate that was placed right in front of the box (Fig 1C). They were required to have the knee and hip in the neutral position in the instance of losing contact with the box. The opposite leg had to bend at the knee to prevent touching the ground. The participants were instructed to stabilize as quickly as possible and remain stable for ~10 s. The trial was repeated if participants lost balance and touched the floor with the opposite leg. Three trials were performed for each leg in an alternating order, with 60 s breaks between the trials.

### Equipment and data processing

A piezoelectric force platform (model 9260AA, Kistler, Winterthur, Switzerland) was used to collect the ground reaction force data (sampling rate: 1000 Hz) for both tasks. The data was automatically filtered (low-pass Butterworth, 2^nd^ order, 10 Hz) in the MARS Software (version 4.0, Kistler, Winterthur, Switzerland). The data was further automatically processed in MARS to obtain the outcome variables of interest. For all the outcome variables, the average of the three trials was used for further analyses.

In terms of quiet stance body sway, we considered the mean CoP velocity (total, anterior-posterior (AP) and medial-lateral (ML)), CoP amplitude (AP and ML), CoP area and CoP frequency (AP and ML). The CoP velocity was defined as the length of the trajectory of the COP sway divided by the measurement time. The CoP amplitude was defined as the average amount of the CoP sway in AP or ML direction, calculated as the common length of the trajectory of the COP sway only in the given direction, divided by the number of changes of movement direction. The CoP area was defined as the area of the ellipse fitted over the COP trajectory so that it contains 100% of all the data points. The CoP frequency was defined as the frequency of the oscillations of the CoP calculated as the number of peaks in AP or ML direction (i.e. changes in direction of CoP movement) divided by the measurement time.

For the landing task, time to stabilization (TTS) and dynamic postural stability index (DPSI) were considered. TTS were analyzed by using the method described by Colby et al. [25] and Wikstrom et al. [26] We determined the TTS based on the total, ML and AP CoP movement by sequential estimation. In short, an algorithm is used to calculate a cumulative average of the data points in a series, by adding in points at a time in succession. Then, the cumulative average is compared with the overall mean of the series, and the series is considered to be stable when the sequential average remained within 0.25 of the standard deviation of the overall mean. Vertical force based TTS was determined as the time when the vertical force reached and stayed within 5% of the participants’ body weight. In addition, we calculated the DPSI and its components (AP stability index (APSI), ML stability index (MLSI) and vertical stability index (VSI), following the methodology outlined by Wikstrom et al. [26]. All indexes were calculated for 3 s and 5 s time windows after the landing.

### Determination of leg preference and dominance

Leg preference was determined a priori. In the first approach, the preferred leg was determined as the self-reported leg preferably used to kick a ball. In the second approach, the preferred leg was determined as the self-reported leg preferably used for single-leg vertical jumping. Leg dominance was determined post hoc, based on the observed performance in body sway and landing tasks. Two approaches were used, one based on the total CoP velocity in the body sway tasks, and one based on the DPSI in the landing task. Since lower values of CoP velocity and DPSI both represent superior balance ability, the dominant leg was determined as the leg with lower CoP velocity or DPSI.

### Statistical analysis

The statistical analyses were done in SPSS Software (version 25.0). Descriptive statistics is presented as means ± standard deviation. The normality of the data distribution was assured with Shapiro-Wilk’s test and equality of variances with Levene’s test. Pairwise t-tests were used to assess the differences between the dominant/preferred and non-dominant/non-preferred leg. These analyses were conducted once for each leg categorization approach. Mixed-moderal ANOVA with gender as between-subject factor and leg as within subject factor was conducted to explore the interaction between leg preference and gender. Furthermore, independent sample t-tests were used to assess differences between genders for each leg separately. Cohen’s d was used to determine the effect sizes, and was interpreted as trivial (0.0–0.1), small (0.1–0.3), medium (0.4–0.7), large (0.8–1.4) and very large (> 1.4). The threshold for statistical significance was set at p < 0.05.

## Results

### Effects of leg preference

Table 1 summarizes the differences between preferred and non-preferred legs based on the preference for kicking a ball. Statistically significant differences between the legs were found for CoP AP velocity (p = 0.017) and CoP AP amplitude (p < 0.001), as well as CoP frequency in both AP and ML directions (p = 0.001–0.002). Specifically, CoP velocity and amplitude were smaller in the preferred leg, while the opposite was true for the CoP frequency. The effect sizes were small (d = 0.17 ≤ d ≤ 0.28). For the landing task, CoP ML TTS was statistically significantly (p = 0.012) shorter for the preferred leg (0.28 ± 0.38 s) compared to the non-preferred leg (0.47 ± 0.60 s) (d = 0.38). No differences between the legs were observed for the rest of the outcome variables.

**Table 1:** Effect of leg preference (for kicking a ball) on body sway and landing variables.

Task	Outcome variable	Preferred leg		Non-preferred leg		Difference		
		Mean	SD	Mean	SD	t	p	ES
Single-leg stance 30 s	CoP VEL—total [mm/s]	43.02	9.20	43.67	10.11	1.15	0.253	0.07
	CoP VEL—AP [mm/s]	26.29	6.28	27.40	7.14	2.44	0.017	0.17
	CoP VEL–ML [mm/s]	28.56	6.21	28.37	6.50	-0.46	0.644	0.03
	CoP AMP—AP [mm]	5.92	1.49	6.39	1.80	3.72	0.000	0.28
	CoP AMP–ML [mm]	8.45	2.04	8.64	2.29	1.14	0.259	0.09
	CoP Area [mm^2]	178.8	56.9	173.1	51.5	-1.54	0.128	0.11
	CoP FRQ—AP [Hz]	4.52	0.62	4.38	0.63	-3.41	0.001	0.22
	CoP FRQ–ML [Hz]	3.44	0.46	3.35	0.48	-3.11	0.002	0.20
Single leg landing	Time to stabilization—VF [s]	0.57	0.17	0.57	0.15	0.146	0.884	0.02
	Time to stabilization—CoP [s]	1.90	0.72	1.76	0.67	-1.56	0.122	0.20
	Time to stabilization—CoP—AP [s]	1.06	0.96	1.24	0.87	1.475	0.144	0.19
	Time to stabilization—CoP—ML [s]	0.28	0.38	0.47	0.60	2.564	0.012	0.38
	APSI—3 seconds [a.u.]	0.10	0.05	0.10	0.03	-1.05	0.297	0.08
	MLSI—3 seconds [a.u.]	0.04	0.02	0.03	0.02	-1.29	0.200	0.10
	VSI—3 seconds [a.u.]	0.50	1.25	0.42	1.02	-1.05	0.299	0.07
	DPSI—3 seconds [a.u.]	0.52	1.25	0.44	1.02	-1.04	0.300	0.07
	APSI—5 seconds [a.u.]	0.08	0.04	0.08	0.03	-1.05	0.295	0.08
	MLSI—5 seconds [a.u.]	0.03	0.02	0.03	0.01	-1.39	0.167	0.11
	VSI—5 seconds [a.u.]	0.43	1.23	0.35	1.01	-1.04	0.304	0.07
	DPSI—5 seconds [a.u.]	0.44	1.23	0.36	1.01	-1.03	0.304	0.07

CoP–center of pressure; VEL–velocity; AMP–amplitude; FRQ–frequency, AP–anterior-posterior; ML–medial-lateral; VF–vertical force; APSI–anterior-posterior stability index; MLSI–medial-lateral stability index; VSI–vertical stability index; DPSI–dynamic postural stability index.

Table 2 summarizes the differences between preferred and non-preferred leg for jumping. None of the body sway parameters was statistically significantly different between the legs, and all effect sizes were trivial or small (d < 0.09). Similarly, none of the landing parameters was different between groups (d < 0.14).

**Table 2:** Effect of leg preference (for single-leg jumping) on body sway and landing variables.

Task	Outcome variable	Preferred leg		Non-preferred leg		Difference		
		Mean	SD	Mean	SD	t	p	ES
Single-leg stance 30 s	CoP VEL—total [mm/s]	43.38	9.72	43.67	10.11	0.14	0.889	0.03
	CoP VEL—AP [mm/s]	26.93	6.75	27.40	7.14	0.35	0.724	0.07
	CoP VEL—ML[mm/s]	28.46	6.40	28.37	6.50	-0.06	0.956	0.01
	CoP AMP—AP [mm]	6.22	1.67	6.39	1.80	1.05	0.296	0.09
	CoP AMP—ML[mm]	8.62	2.26	8.64	2.29	0.96	0.340	0.01
	CoP Area [mm^2]	174.8	54.1	173.1	51.5	-0.65	0.519	0.03
	CoP FRQ—AP [Hz]	4.41	0.61	4.38	0.63	-1.89	0.062	0.05
	CoP FRQ–ML [Hz]	3.37	0.48	3.35	0.48	-1.93	0.056	0.03
Single leg landing	Time to stabilization—VF [s]	0.57	0.15	0.58	0.17	-0.29	0.771	0.04
	Time to stabilization—CoP [s]	1.83	0.69	1.83	0.71	-0.01	0.996	0.00
	Time to stabilization—CoP—AP [s]	1.22	0.90	1.09	0.94	1.06	0.291	0.14
	Time to stabilization—CoP—ML [s]	0.37	0.46	0.37	0.57	0.01	0.990	0.00
	APSI—3 seconds [a.u.]	0.10	0.03	0.10	0.05	-0.31	0.756	0.02
	MLSI—3 seconds [a.u.]	0.03	0.02	0.04	0.02	-1.79	0.076	0.14
	VSI—3 seconds [a.u.]	0.42	1.02	0.50	1.25	-1.05	0.297	0.07
	DPSI—3 seconds [a.u.]	0.44	1.02	0.52	1.25	-1.04	0.302	0.07
	APSI—5 seconds [a.u.]	0.08	0.03	0.08	0.04	-0.29	0.774	0.02
	MLSI—5 seconds [a.u.]	0.03	0.01	0.03	0.02	-1.65	0.102	0.13
	VSI—5 seconds [a.u.]	0.35	1.01	0.43	1.23	-1.04	0.301	0.07
	DPSI—5 seconds [a.u.]	0.36	1.01	0.44	1.23	-1.03	0.305	0.07

CoP–center of pressure; VEL–velocity; AMP–amplitude; FRQ–frequency, AP–anterior-posterior; ML–medial-lateral; VF–vertical force; APSI–anterior-posterior stability index; MLSI–medial-lateral stability index; VSI–vertical stability index; DPSI–dynamic postural stability index.

### Effects of leg dominance

Table 3 summarizes the comparison between the legs, classified as dominant and non-dominant based on CoP total velocity. There were statistically significant differences (better balance ability in the dominant leg) between the legs in all CoP velocity and amplitude variables in body sway (all p < 0.001; 0.41 ≤ d ≤ 0.45). There were no differences in terms of CoP area and frequency. Based on this classification, there were also differences in APSI and MLSI during landing (both in 3 s and 5 s windows), however, in this case, the dominant leg performed worse (p < 0.001, d = 0.84 ≤ d ≤ 1.01).

**Table 3:** Effect of leg dominance (based on total center of pressure velocity during quiet stance) on body sway and landing variables.

Task	Outcome variable	Dominant leg		Non-dominant leg		Difference		
		Mean	SD	Mean	SD	t	p	ES
Single-leg stance 30 s	CoP VEL—total [mm/s]	41.19	8.78	45.47	10.05	-12.2	0.000	0.45
	CoP VEL—AP [mm/s]	25.40	5.88	28.28	7.24	-8.0	0.000	0.44
	CoP VEL—ML[mm/s]	27.18	5.92	29.75	6.50	-8.4	0.000	0.41
	CoP AMP—AP [mm]	5.81	1.48	6.50	1.80	-6.2	0.000	0.42
	CoP AMP—ML[mm]	8.07	1.83	9.03	2.39	-7.4	0.000	0.45
	CoP Area [mm^2]	172.9	57.9	179.0	50.4	-1.6	0.106	0.11
	CoP FRQ—AP [Hz]	4.45	0.64	4.44	0.65	0.2	0.844	0.01
	CoP FRQ—ML[Hz]	3.44	0.47	3.37	0.50	1.6	0.116	0.13
Single leg landing	Time to stabilization—VF [s]	0.57	0.18	0.63	0.35	-1.4	0.174	0.19
	Time to stabilization—CoP [s]	1.77	0.59	1.88	0.82	-1.3	0.207	0.16
	Time to stabilization—CoP—AP [s]	1.20	0.98	1.07	0.93	1.0	0.308	0.14
	Time to stabilization—CoP—ML [s]	0.39	0.53	0.32	0.55	0.8	0.400	0.13
	APSI—3 seconds [a.u.]	0.10	0.04	0.05	0.06	6.6	0.000	1.01
	MLSI—3 seconds [a.u.]	0.03	0.02	0.02	0.02	5.6	0.000	0.86
	VSI—3 seconds [a.u.]	0.42	1.01	0.35	1.29	0.8	0.398	0.06
	DPSI—3 seconds [a.u.]	0.44	1.01	0.36	1.29	0.9	0.346	0.07
	APSI—5 seconds [a.u.]	0.08	0.03	0.04	0.04	6.6	0.000	1.00
	MLSI—5 seconds [a.u.]	0.03	0.01	0.01	0.02	5.6	0.000	0.84
	VSI—5 seconds [a.u.]	0.35	1.01	0.31	1.27	0.5	0.629	0.03
	DPSI—5 seconds [a.u.]	0.36	1.01	0.32	1.27	0.6	0.573	0.04

CoP–center of pressure; VEL–velocity; AMP–amplitude; FRQ–frequency, AP–anterior-posterior; ML–medial-lateral; VF–vertical force; APSI–anterior-posterior stability index; MLSI–medial-lateral stability index; VSI–vertical stability index; DPSI–dynamic postural stability index.

When the leg dominance was determined based on DPSI (Table 4), there were no differences between the legs in any of the body sway variables. Statistically significant differences between the legs were found for MLSI (3 and 5 s windows; p = 0.017–0.021; d = 0.18). Interestingly, even the DPSI itself, which was used to determine the leg dominance, was not statistically significantly different between the legs (p = 0.215–0.233; 0.08 ≤ d ≤ 0.09).

**Table 4:** Effect of leg dominance (based on dynamic postural stability index) on body sway and landing variables.

Task	Outcome variable	Dominant leg		Non-dominant leg		Difference		
		Mean	SD	Mean	SD	t	p	ES
Single-leg stance 30 s	CoP VEL—total [mm/s]	43.78	9.66	42.91	9.67	1.6	0.122	0.09
	CoP VEL—AP [mm/s]	27.17	6.75	26.51	6.72	1.4	0.160	0.10
	CoP VEL–ML [mm/s]	28.73	6.30	28.21	6.40	1.3	0.201	0.08
	CoP AMP—AP [mm]	6.26	1.73	6.05	1.60	1.6	0.110	0.13
	CoP AMP–ML [mm]	8.60	2.07	8.48	2.26	0.7	0.468	0.06
	CoP Area [mm^2]	176.06	50.11	175.98	58.35	0.0	0.983	0.00
	CoP FRQ—AP [Hz]	4.43	0.62	4.47	0.64	-0.7	0.462	0.05
	CoP FRQ–ML [Hz]	3.40	0.48	3.40	0.47	0.0	0.989	0.00
Single leg landing	Time to stabilization—VF [s]	0.57	0.15	0.57	0.17	-0.2	0.828	0.03
	Time to stabilization—CoP [s]	1.77	0.64	1.89	0.75	-1.4	0.156	0.18
	Time to stabilization—CoP—AP [s]	1.23	0.95	1.08	0.89	1.3	0.209	0.17
	Time to stabilization—CoP—ML [s]	0.38	0.50	0.36	0.53	0.2	0.828	0.03
	APSI—3 seconds [a.u.]	0.10	0.04	0.10	0.04	-1.1	0.259	0.08
	MLSI—3 seconds [a.u.]	0.03	0.02	0.04	0.02	-2.3	0.021	0.18
	VSI—3 seconds [a.u.]	0.41	1.01	0.51	1.25	-1.3	0.213	0.09
	DPSI—3 seconds [a.u.]	0.43	1.01	0.52	1.25	-1.2	0.215	0.09
	APSI—5 seconds [a.u.]	0.08	0.03	0.08	0.03	-1.2	0.246	0.08
	MLSI—5 seconds [a.u.]	0.03	0.01	0.03	0.02	-2.4	0.017	0.18
	VSI—5 seconds [a.u.]	0.34	1.01	0.43	1.23	-1.2	0.231	0.08
	DPSI—5 seconds [a.u.]	0.36	1.01	0.45	1.23	-1.2	0.233	0.08

CoP–center of pressure; VEL–velocity; AMP–amplitude; FRQ–frequency, AP–anterior-posterior; ML–medial-lateral; VF–vertical force; APSI–anterior-posterior stability index; MLSI–medial-lateral stability index; VSI–vertical stability index; DPSI–dynamic postural stability index.

### Effects of gender

There were no gender × leg interactions in any of the outcomes from static and dynamic balance tasks (all p ≤ 0.433), regardless of the approach to leg classification. In terms of static balance task and using preference, females had smaller CoP ML velocity (p = 0.018; d = 0.48 on preferred and 0.50 on the non-preferred leg) as well as CoP ML amplitude (p = 0.006; d = 0.52 on preferred and 0.58 on the non-preferred leg). There were no statistically significant main effects of gender in landing outcome variables (d < 0.22). The descriptive statistics calculated for each gender separately is available in S1 File.

## Discussion

The purpose of this study was to explore the differences between the preferred and non-preferred legs in single-leg body sway test and single-leg landing test in a sample of tennis players. Contrary to our hypothesis, more variables showed differences between the legs in static balance tasks compared to the landing task. Also contrary to our hypothesis, the approach to classification of the legs based on the participant’s preference for single-leg jumping task did not lead to the detection of larger differences between the legs. In addition, single-leg quiet stance body sway appears to be more sensitive to leg preferences than the landing task. As expected, larger effect sizes for inter-leg differences were observed when dominance was considered. However, this was evident only in the static task, suggesting that static balance is characterized by a larger limb dominance than the dynamic balance.

### Effects of leg preference

It is well documented that each of the upper limbs is specialized for a specific task. Namely, the limb that is usually referred to as the preferred (based on the preference for writing) is specialized for object manipulation (mobility), while the opposite limb is specialized for controlling position (stability) [27]. Considering static balance tasks, it was observed that after single-leg balance training, there is a noticeable transfer to the untrained leg, and that the magnitude of this transfer is direction-dependent (i.e. higher in the right-to-left direction than vice versa) [28], indicating a potential asymmetry in balance control. Evidence from studies on stroke patients suggest that right cerebral hemisphere has a more prominent role in balance control [29, 30]. Considering the asymmetries observed in the upper limb and the prominent role of right hemisphere in balance, one would expect the non-dominant/non-preferred leg to exhibit a better static balance compared to the dominant/preferred leg. However, our results indicate that the differences between the legs, albeit small and confined to only a limited number of variables, favor the dominant/preferred leg. Previous studies that investigated this issue found either no differences between the legs regarding postural balance [5] or superior results for the preferred leg to kick a ball [16, 17]. Overall, the evidence tends to show no systematic difference between the legs, as summarized in the recent review [13]. Moreover, even for the variables that differed between the legs, the effect sizes were small, as observed in our previously published study [17]. Herein, we also showed that the differences between the legs are small in terms of transient behavior of the postural sway (i.e. the changes of body sway metric throughout the trial) [17]. In sum, the leg preference is unlikely to notably influence the analysis of postural balance, even when the goal is to compare the legs (e.g. as in the comparison of injured and uninjured legs).

The absence of the differences between the legs could imply that the classification (of the legs) itself is inappropriate. Indeed, our classifications were based on manipulative and locomotor tasks, which are different than the tasks used to study postural balance. Perhaps, different methods of classification could be used to classify the legs. For the hands, it was shown that the side-specific specialization is related to functional hemispheric asymmetries [27, 31], which were also implicated for lower legs [32]. However, classifying the legs based on the handedness would likely not have affected the results, as it was reported that in 96% of cases, the side of the preferred upper arm is matched with the side of the preferred leg for kicking a ball [16]. Thus, it currently remains unknown if there could be a reasonable method of the determination of the leg preference that would show consistent differences between the preferred and non-preferred legs. It is also difficult to explain why the body sway, but not the landing task, showed statistically significant differences between the legs. The most likely explanation is that higher within- and between-participant variability in landing task variables prevented more statistically significant outcomes. Moreover, a very high task-specificity is evident across balance tasks [12].

### Effects of leg dominance

**Fig 2:**
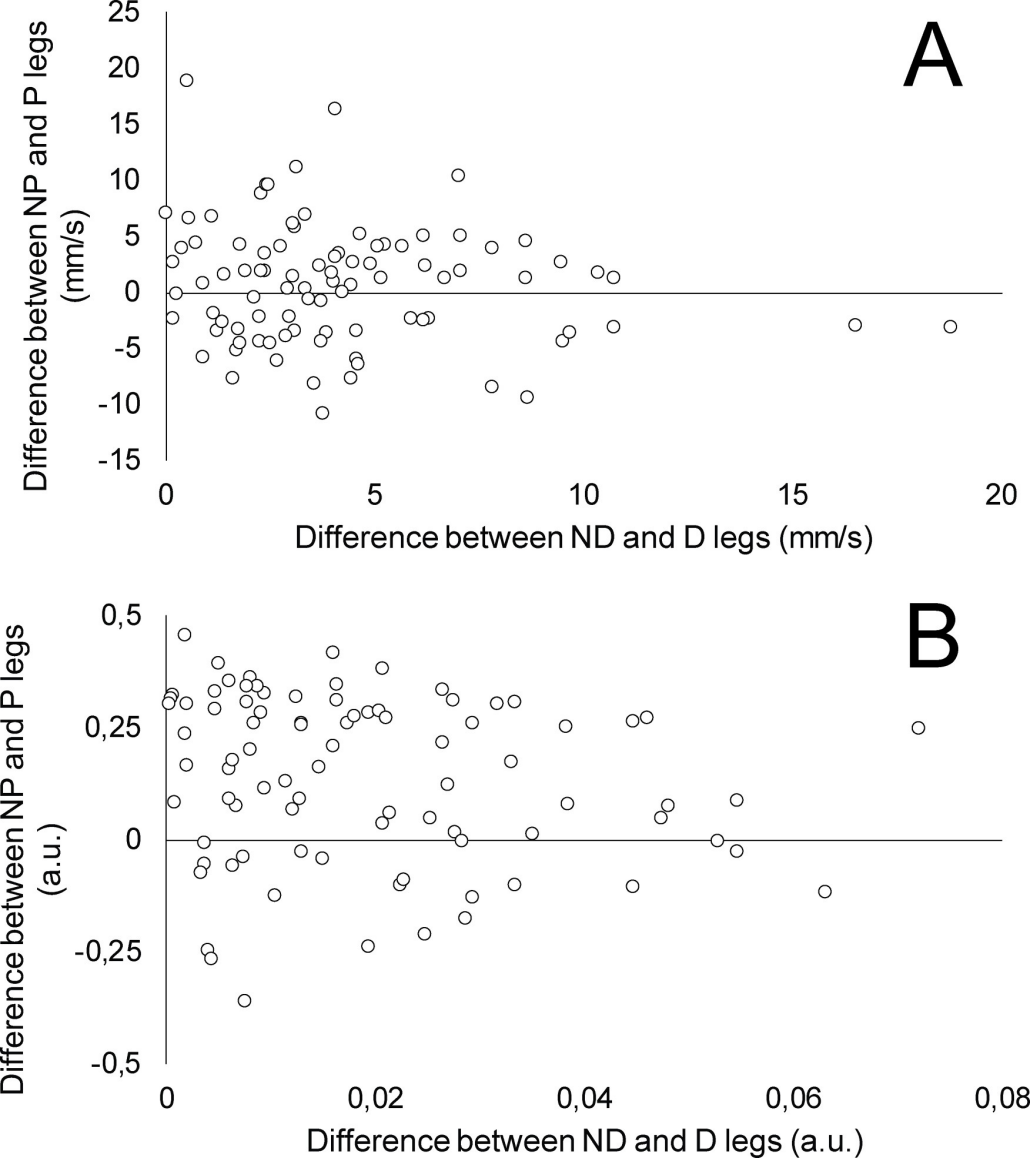
The relationship between the differences between the legs based on preference and dominant for CoP velocity during static balance task (A) and dynamic postural stability index B) in the dynamic task. D–dominant; ND–non dominant; P–preferred; NP–non-preferred.

As expected, the effects of leg dominance were larger compared to leg preference, most notably for static balance (i.e., body sway variables). This is inevitable because the performance variables assessed during the task itself (CoP velocity and DPSI in our study) are used to determine the dominance. The observed effects of leg dominance demonstrate that meaningful differences between the legs do exist, however, they are not directly related to leg preference. This is also shown in Fig 2, wherein the differences between dominant and non-dominant (x-axis) and preferred and non-preferred (y-axis) are plotted. This means that if we are comparing inter-leg differences in postural balance when comparing injured and uninjured legs, baseline (pre-injury) data for both legs is useful to ensure that the leg dominance is not confounding the comparison. In other words, the data from the uninjured leg cannot be reliably used as a reference without baseline data. Another interesting finding was that the dominant leg, determined according to CoP velocity during static task, showed worse APSI and MLSI. This suggests that leg dominance is not consistent across the two tasks. This is in accordance with previous studies that have shown that there is little common variance between different balance tasks [12]. It should be noted that APSI and MLSI are less reliable variables than DPSI, which was not different between the dominant and non-dominant legs (based on CoP velocity determination).

### Gender differences

On a secondary note, we investigated the effects of gender and observed better postural balance in females compared to males, but only in static balance. Larger body sway in males has been observed before [33], although a recent synthesis of evidence suggests that the gender differences are inconsistent in children and youth [34]. In terms of landing, a previous study [20] has shown slightly worse postural balance in females, although the differences were not substantial (i.e., DPSI normalized to potential energy in males: 0.005 ± 0.001 and females 0.006 ± 0.001). Our results, on the contrary, showed no differences between the genders. Since a substantial difference in kinematic patterns of landing between genders is evident [35, 36], it is questionable whether the variables related to postural balance should even be used for gender comparisons.

### Limitations

The limitations of the present study should be acknowledged. The major limitation of the study is the inclusion of only tennis players, which makes the generalization to other population very limited. We did not record any kinematical data or muscle activity, which could reveal differences between the legs. For instance, differences in kinematic patterns of landing could be present. Even in static balance task, different strategies in task execution between the legs might be present (reflected in different kinematic patterns and/or muscle activity), and not necessarily reflected in CoP movements [37]. Future studies should consider the differences in kinematic patterns of landing between the preferred and non-preferred leg. Another drawback of the study was the slight discrepancy between the genders regarding age (males: 18.4 ± 14.8 years, females: 16.2 ± 2.68 years).

## Conclusion

This study showed that small differences in static balance may exist between the preferred and non-preferred leg when the preference is determined based on the manipulation task (kicking a ball). No differences were found when the classification was carried out based on the preference for executing a single-leg jump. Furthermore, no differences were found between the legs in terms of the dynamic balance (landing task). Thus, leg preference plays a small role in the assessment of static balance. However, moderate effects of limb dominance are present in body sway variables, which implies that caution is nonetheless needed when comparing the legs. In sum, the distinction of preference and dominance is important, and we urge future researchers to pay careful attention to the nomenclature.

## Supporting information

S1 File Gender specific descriptive statistics. (XLSX)
